# Maternal respectful care and Post-Traumatic Stress Disorder among postpartum mothers in Israel

**DOI:** 10.1093/eurpub/ckac130.045

**Published:** 2022-10-25

**Authors:** S Abu-Hamad, N Daoud

**Affiliations:** School of Public Health, Ben Gurion University of the Negev, Beer Sheva, Israel

## Abstract

**Background:**

Maternal Respectful Care (MRC) approach was recommended by the WHO for improving maternal birth experience and mental health. We examined the association between MRC and Post-Traumatic Stress Disorder (PTSD) among postpartum women.

**Methods:**

A cross-sectional study took place between November 2020 and October 2021. 817 postpartum women (Jewish- 444 Arab-373) were interviewed via Zoom due to Covid-19 limitations. MRC was measured by 26 statements from disrespectful/ abusive care during childbirth in facilities (DACF); 4 statements from the Mother on Respect questionnaire (MOR-feeling mistreatment based on ethno-national background, level of command of the Hebrew language and disagreement with the staff); and 4 statements regarding abuse of the NorVold Abuse Questionnaire (NorAQ). PTSD was measured by Solomon et al.'s tool, and dichotomized into 12 score cutoff. (Yes /No PTSD).

**Results:**

PTSD prevalence was 14.3%, significantly higher among Palestinian-Arab compared to Jewish women (22.0%,7.9%, respectively), and it was higher in women who had lower MRC scores: did not receive postpartum education (19.9%,11.9 % respectively) received; received midwife support 11.7%,18.4% didn't receive; reported racism 26.1%,11.4% not; Felt humiliated at healthcare services vs. not (16.8% , 10.6%, respectively); women who reported mistreatment based on national/cultural background, compared to others (MOR) (36.6%,13.1%, respectively). In the multivariate analysis after adjusting to different independent variables, Palestinian-Arab women were 6.04 times at risk for PTSD (OR = 6.04,95% CI = 3.38-10.78),Women who reported racism are 2.14 times more likely to PTSD (OR = 2.14, 95% CI = 1.30-3.54). Women who reported feeling humiliated visiting the health care system are 2.08 times more likely to PTSD (OR = 2.08,95% CI = 1.23-3.52).

**Conclusions:**

MRC is an important factor for maternal PTSD among postpartum women and it should be considered specifically among minority women.

**Key messages:**

- MRC can contribute to quality care, improving birthing mothers experience and reducing negative health outcomes such as PTSD, and it should be improved for women.
- The health care services need to run a program to overcome racism and reduce unrespectful care for postpartum women.

